# Virtual Reality Aided Therapy towards Health 4.0: A Two-Decade Bibliometric Analysis

**DOI:** 10.3390/ijerph19031525

**Published:** 2022-01-28

**Authors:** Zhen Liu, Lingfeng Ren, Chang Xiao, Ke Zhang, Peter Demian

**Affiliations:** 1 School of Design, South China University of Technology, Guangzhou 510006, China; liuzjames@scut.edu.cn (Z.L.); kezh@scut.edu.cn (K.Z.); 2 School of Architecture, Building and Civil Engineering, Loughborough University, Loughborough LE11 3TU, UK; p.demian@lboro.ac.uk

**Keywords:** health care, virtual reality, therapy, VOSviewer, bibliometric, post-traumatic stress disorder (PTSD), anxiety and fear related disorder (A&F), diseases of the nervous system (DNS), pain management, Health 4.0, Health Metaverse

## Abstract

Health 4.0 aligns with Industry 4.0 and encourages the application of the latest technologies to healthcare. Virtual reality (VR) is a potentially significant component of the Health 4.0 vision. Though VR in health care is a popular topic, there is little knowledge of VR-aided therapy from a macro perspective. Therefore, this paper was aimed to explore the research of VR in aiding therapy, thus providing a potential guideline for futures application of therapeutic VR in healthcare towards Health 4.0. A mixed research method was adopted for this research, which comprised the use of a bibliometric analysis (a quantitative method) to conduct a macro overview of VR-aided therapy, the identification of significant research structures and topics, and a qualitative review of the literature to reveal deeper insights. Four major research areas of VR-aided therapy were identified and investigated, i.e., post-traumatic stress disorder (PTSD), anxiety and fear related disorder (A&F), diseases of the nervous system (DNS), and pain management, including related medical conditions, therapies, methods, and outcomes. This study is the first to use VOSviewer, a commonly used software tool for constructing and visualizing bibliometric networks and developed by Center for Science and Technology Studies, Leiden University, the Netherlands, to conduct bibliometric analyses on VR-aided therapy from the perspective of Web of Science core collection (WoSc), which objectively and visually shows research structures and topics, therefore offering instructive insights for health care stakeholders (particularly researchers and service providers) such as including integrating more innovative therapies, emphasizing psychological benefits, using game elements, and introducing design research. The results of this paper facilitate with achieving the vision of Health 4.0 and illustrating a two-decade (2000 to year 2020) map of pre-life of the Health Metaverse.

## 1. Introduction

Health is a state of complete physical, mental, and social well-being [1] that has been given particular attention following the establishment of the Millennium Development Goals [2]. In September 2015, the General Assembly of the United Nations adopted the Sustainable Development Goals (SDGs) to ensure healthy lives and promote well-being for all ages [3]. Concurrently, there has been increasing demand for guidance regarding the pathways and resources needed to realize health-related SDGs [4].

Health care (HC) is typically considered a chief determinant to promote the health of people around the world. The share of HC in global revenue has been slowly but steadily increasing in the past few decades, and cross-country evidence has shown that health investment will return substantial health returns [5]. However, there are massive conflicting goals in HC, including accessibility, profitability, quality, cost containment, safety, convenience, patient-centeredness, and satisfaction, thus making it hard to achieve high value [6]. Adopting Health 4.0 in HC may improve HC services and operations, as well as enhance HC systems [7], which is probably the future of HC. Health 4.0 is a strategic concept for the health domain that stemmed from Industry 4.0, aiming to provide real-time personalized medical services for patients and advanced virtualization for HC stakeholders [8]. Health 4.0 has two goals: the provision of high-quality healthcare services and improved efficiency and effectiveness closely considering cost and resource utilization [7]. The vision of Health 4.0 is to achieve the coordination of its three paramount pillars, namely people, technology, and design; therefore, the healthcare system can more consistently and effectively realize the value of data to improve healthcare services and relationships between healthcare stakeholders, thus helping to shift the whole healthcare industry from a passive, service-oriented charging system to a value-based system [9]. The latest technologies are expected to transform HC, while prioritizing the needs of patients since they are the principal stakeholders of the HC system [9].

Virtual reality (VR) is regarded as an information and education tool, as well as a rehabilitation tool for patients [10]. The term refers to a novel technology set, including computers or mobile devices with graphics cards of interactive 3D visualization, controllers, and head-mounted displays (HMDs) embedded with position trackers [11]. As this technology advances, more immersive and satisfactory virtual environments can be created, thus supporting learning, medicine, and HC in overcoming their traditional constraints [12]. VR can provide remarkable improvements to various medical treatments, enabling HC providers to deliver more positive experiences for patients by creating virtual 3D environments [13,14]. In addition, it can assist HC staff in collecting and sharing health data via simulation systems, which can play roles in medical decision making and distance learning [15]. It seems that VR has the potential to be a significant component in the Health 4.0 vision of HC.

Among all applications of VR in healthcare services, the therapy aspect has attracted the most attention. Numerous studies have reported that VR-aided therapy can help patients maintain both physical and mental health. For instance, VR integrated with cognitive behavior therapy (CBT) or exposure therapy (ET) can improve mental illnesses, such as public speaking anxiety [16] and driving phobias [17]. For physical diseases, VR-based sports games have been found to improve upper limb motor function and the daily autonomy of stroke survivors [18]. Additionally, VR can be used as a non-invasive therapy for pain management (PM) [19]. Furthermore, VR can enhance the emotions of lung disease patients, alleviating their anxiety and stress [20]. Encouraging evidence published in articles focusing on various clinical conditions has made clinicians more likely to use VR simulations in their research and clinical trials [15]. Accordingly, VR for therapy has become an increasingly important domain in HC and Health 4.0, and VR-aid therapy has become one of the principal applications of VR [21].

VR-aided therapy has become a popular topic that has attracted plenty of studies. Some reviews have aimed to investigate the status of VR aid therapy, but they have usually adopted the perspective of certain medical conditions, such as rehabilitation [22], mental health disorders [23], psychiatric disorders [24], and drug addiction [25]. Several studies have considered the “big picture” of VR-aided therapy. Javaid and Haleem [14] identified the potential applications of VR technology in the medical field, focusing on VR for medical professions, i.e., medical students and doctors. Yeung et al. [26] analyzed the scientific literature on VR and augmented reality (AR) research in medicine, reviewing commonly discussed themes and medical conditions. Tonkavich et al. [27] conducted a systematic mapping review to categorize the therapeutic use of immersive VR in HC, concentrating on the medical environment and patient population without deeply investigating various therapies. 

Nevertheless, there have been few macro investigations of VR-aided therapy to provide insight for future research. Therefore, this paper is set to conduct a macro research overview of VR-aided therapy, which is aimed to explore the research of VR in aiding therapy, thus providing a potential guideline for futures application of therapeutic VR in healthcare towards Health 4.0.

## 2. Materials and Methods

A mixed research method was adopted for this research, which comprised the use of a bibliometric analysis (a quantitative method) to conduct a macro overview of VR-aided therapy, the identification of significant research structures and topics, and a qualitative review of the literature to reveal deeper insights. Bibliometric methods can enable quantitative analyses of written publications [28]; they enable researchers to base their findings on aggregated bibliographic data produced by other scientists to obtain insights about research structures, social networks, and topical interests [29]. One branch of bibliometrics is science mapping, which aims to visually reveal the structures and dynamic changes in a field of scientific research [30]; it can provide a spatial representation through physical proximity and relative locations to show how disciplines, fields, specialties, and individual papers or authors are related [31]. Hence, science mapping is useful to explore the macro picture of a research field. 

In this paper, two kinds of science mapping methods were employed, namely bibliographic coupling analysis (BCA) and term co-occurrence analysis (TCA). Both were implemented through the VOSviewer software, which is a commonly used tool for constructing and visualizing bibliometric networks, and developed by Center for Science and Technology Studies, Leiden University, the Netherlands. The VOSviewer pays more attention to graphical representation compared to other tools, which is beneficial for displaying large bibliographic maps in an easy-to-understand manner [32]. 

**Figure 1:**
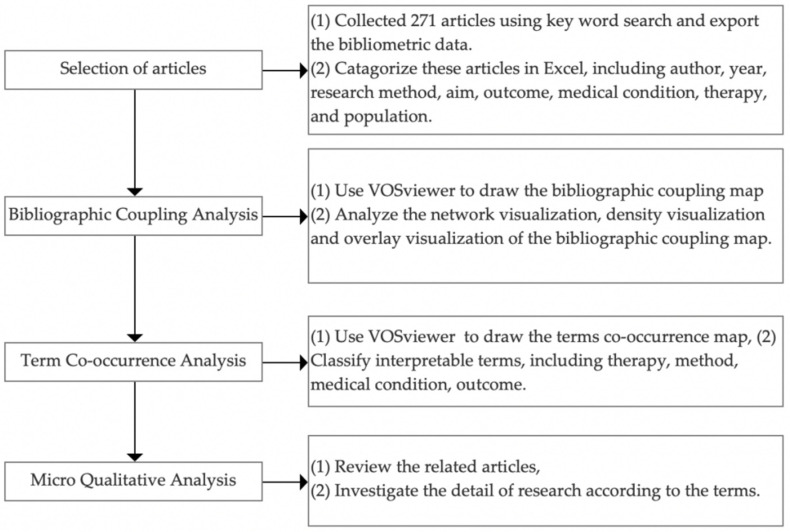
The flow chart of the research methodology.

Figure 1 illustrates the research protocol, which encompasses four stages: (1) the Web of Science core collection (WoSc) was chosen for collecting relevant publications using the query “virtual reality” AND “therapy”. Web of Science is a commonly used database in many disciplines, particularly in the medical and health domain [33], that is suitable for this research. Articles and review articles written in English and published before the year 2021 were selected. The bibliometric data of search results were exported through the export function of WoSc, the record content of which was set to full-record and reference, as well as being tab-delimited (win”UTF-8) for the file format. At the same time, available relevant articles were categorized using Excel. (2) VOSviewer was used to conduct BCA, which clusters publications based on their bibliographies. It is mechanical with no subjective scientific knowledge or judgment of the content [34], and it has a higher accuracy of cluster analysis than other bibliometric methods [35]. (3) A term co-occurrence map was created by VOSviewer and analyzed the occurrence frequency and the common occurrence of terms from the titles and abstracts of the articles. These visual data could be assigned meanings by the researchers to identify latent patterns and pose new questions for further analysis [36]. (4) Interpretable terms from the term co-occurrence map were selected, de-duplicated, and categorized; then, a literature review was conducted based on the two quantitative analyses to obtain more detail data of the field.

## 3. Results

### 3.1. Results of the Bibliographic Coupling Analysis (BCA)

**Figure 2:**
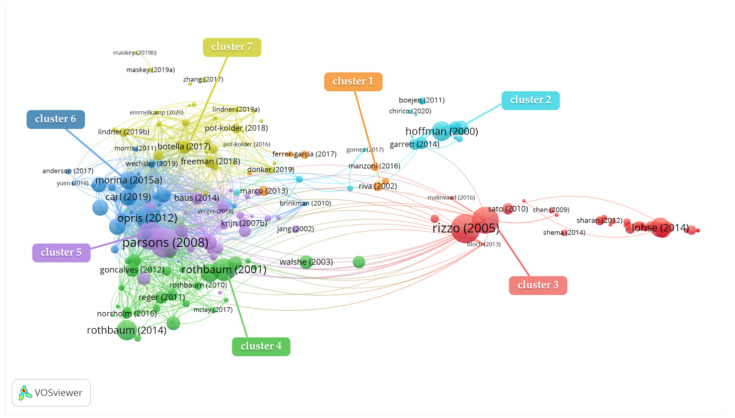
Network visualization of bibliographic coupling analysis of the theme of virtual reality (VR) and therapy in the Web of Science core collection (WoSc) database via VOSviewer (devised and generated by the authors).

**Figure 3:**
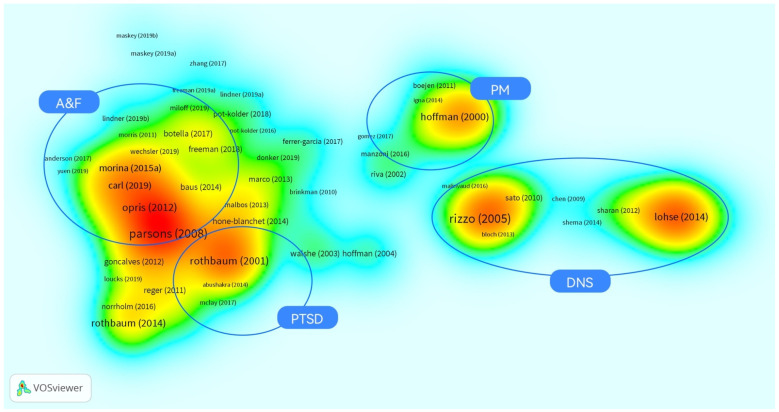
Density visualization of bibliographic coupling analysis of the theme of VR and therapy in the WoSc via VOSviewer (devised and generated by the authors).

**Figure 4:**
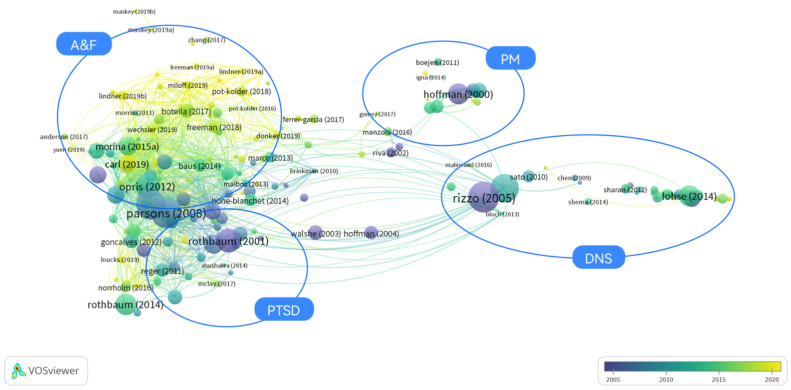
Overlay visualizations of bibliographic coupling analysis of the theme of VR and therapy within two decades (2000 to year 2020) in the WoSc via VOSviewer (devised and generated by the authors).

The VOSviewer was used to generate a bibliographic coupling map of VR and therapy after setting the minimum number of citations of an article to five. Of the total 271 articles, 207 articles met this threshold, and the most extensive set of related articles consisted of 205 articles. In Figure 2, there are circles with text labels (author and year) and lines in the map; every circle with a text label represents an article and the lines represent the relationship between articles. The bibliographic coupling map has three visualization views, namely network visualization (Figure 2), density visualization (Figure 3), and overlay visualization (Figure 4). 

In the network visualization, the size of each circle with a text label is dependent on the numbers of citations of the articles, since citations largely represent the influence of an article [37], and the distance between circles stands for the affinity of articles [38]. As shown in Figure 2, VOSviewer divided these articles into seven clusters with different colors.

Table 1 lists the representative articles of each cluster, showing the authors, years, research methods, research subjects, medical conditions, and therapies. Each cluster has been named in line with the 11th revision of the International Classification of Diseases (ICD-11) provided by International Health Organization [39]. Cluster1 concerns eating disorders (ED); Cluster2 is mainly focused on pain management (PM); Cluster3 concerns diseases of the nervous system (DNS); Cluster4 looks into post-traumatic stress disorder (PTSD); and Cluster5, Cluster6, and Cluster7 are associated with anxiety and fear related disorder (A&F).

In the density visualization (Figure 3), the colors denote the number of nearby articles and their weights [38]. The more an area has been intensively researched, the closer the color is to red. Only four areas, namely A&F, DNS, PTSD, and PM, have formed relatively intensive research areas, as marked in Figure 3. In addition, the density view shows the affinity of research areas through positional distance. As shown in Figure 3, PTSD research is close to A&F, while the PM and DNS research areas are relatively independent and far away from PTSD and A&F. This is consistent with ICD-11 [39] since both PTSD and A&F belong to mental, behavioral, or neurodevelopmental disorders, whilst DNS and PM are related to neurology and rehabilitation.

In the overlay visualization (Figure 4), the color of articles, which transitions from purple to yellow, is determined by the publication year. The older the research is, the closer the color is to purple, and the newer the research is, the closer color is to yellow. Since the earliest article meeting the threshold is the article with the text label of ‘Hoffman (2000)’ [40] indicating the first published VR and therapy study in year 2000, Figure 4 shows the 20-year development of VR-aided therapy from 2000 to year 2020. It was found that the earliest applications in last two decades of VR for therapy were in PTSD [41], PM [40], and DNS [42]. However, recent studies have been more focused on A&F, as shown by the distribution of yellow in Figure 4.

**Figure 5:**
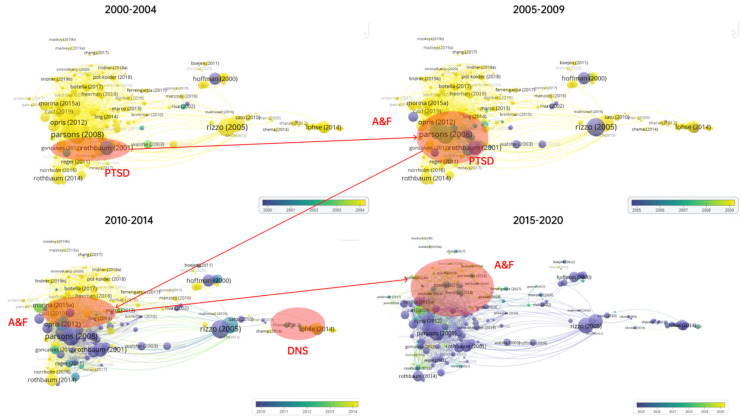
Four ranges in two decades (2000 to year 2020) of overlay visualizations of bibliographic coupling analysis of the theme of VR and therapy in the WoSc via VOSviewer (devised and generated by the authors).

VOSviewer allows users to set the color range of articles. The four limited year ranges have been set, i.e., from 2000 to 2004, 2005 to 2009, 2010 to 2014, and 2015 to 2020, to explore the chronological development of VR-aided therapy. In the virtualization of the four ranges, as shown in Figure 5, yellow or purple stands for the research beyond the specified range and green represents contemporary research of the specified year range. It was found that the focus of research has changed over the long research period: VR-aided therapy was mainly used to treat PTSD in the first five years of this century, i.e., from 2000 to 2004; in the following five years, studies began to shift from PTSD to A&F; from 2010 to 2014, the studies were mainly distributed in two fields of A&F and DNS; and in the most recent five years, the majority of the studies concentrated on A&F.

### 3.2. Results of Terms Co-Occurrence Analysis (TCA)

For this section, the authors used VOSviewer to extract the terms from the titles and abstracts of collected articles, and then they calculated their occurrences and co-occurrences using the binary counting method. Regarding occurrences, Table 2 lists the 10 most influential terms in virtual reality-aided therapy via VOSviewer: virtual reality exposure therapy, exposure therapy, virtual reality therapy, test, PTSD, rehabilitation, control group, anxiety disorder, post-traumatic stress disorder, and phobia. 

**Figure 6:**
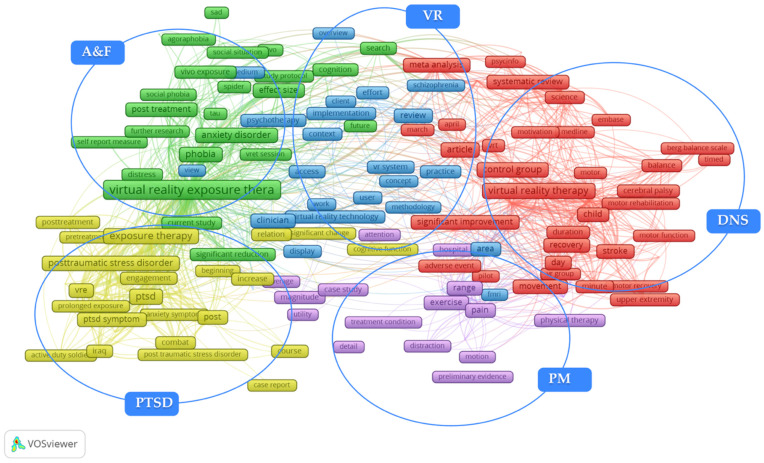
Network visualization of term co-occurrence analysis of the theme of VR and therapy within two decades (2000 to year 2020) in the WoSc database via VOSviewer (devised and generated by the authors).

During the TCA, the minimum occurrences of terms has been set to five. Out of the 6497 terms, 393 met the threshold, and VOSviewer selects 60% of the most relevant terms, thus resulting in 236 terms used to create the term co-occurrence map of VR-aided therapy, as shown in Figure 6. Similar to Figure 2, Figure 6 shows circles with text labels and lines, where a circle with a text label represents a term and a line represents the relationship between terms. VOSviewer divided these terms into five clusters, as marked in Figure 6. There is a cluster in the middle of the map mainly related to technology, including the terms virtual reality technology, VR system, and fMRI, which is surrounded by four clusters corresponding to the four areas determined in the density visualization of BCA (Figure 3), namely PTSD, A&F, DNS, and PM. Figure 6 shows that the link between A&F and PTSD is strong, but they are rarely directly associated with DNS and PM.

To explore four application areas of VR-aided therapy in more detail, all terms in Figure 6 have been scrutinized and selected the explanatory significance terms. Then, they amalgamated the terms with same meanings, such as virtual reality therapy and VRT. It was found that there were four kinds of terms in each area, specifically, there were terms about medical conditions, therapies, research methods, and outcomes. To put it more clearly, the medical condition terms refer to the illness, symptoms, and patients, such as PTSD. The therapy terms refer to the treatments that were used in articles, such as virtual reality exposure therapy. The outcome terms refer to the therapeutic effects and other benefits, such as the term significant change. The terms that come from the title and abstract of the articles are classified in the tables in the following sections. These terms provide more specific knowledge, compared to BCA, and aid in the deep exploration of the research content of this field to identify a micro qualitative analysis pattern. The full classification of terms is presented in the following sections, which consider micro qualitative analysis.

### 3.3. Micro Qualitative Analysis

For this section, the results from the previous two quantitative analyses have been integrated and explored deeper based on related articles. Specifically, four major applications of VR-aided therapy have been identified through the bibliographic analysis in Section 3.1, namely PTSD, A&F, DNS, and PM. Then, in Section 3.2, term occurrence analysis has been used to map out the terms of each application and found that there were four categories, namely therapy, research method, medical condition, and outcome. To align with aforementioned results, the qualitative analysis was divided to four parts corresponding to four major applications. For each part, the classification of terms in Figure 6 has been shown first, which reflect the partial details, followed by a further review of related articles. This review is supported by the quantitative analyses, for which it is supplementary.

#### 3.3.1. Post-Traumatic Stress Disorder (PTSD)

Post-traumatic stress disorder is perhaps the most common psychiatric disorder to occur after an encounter with a traumatic event, and it can result in a major public health burden because of inefficient universal treatment [43]. Terms associated with PTSD from Figure 6 are categorized in Table 3.

The terms of medical conditions, e.g., IRAP, combat, veteran, and active duty soldier, in Table 3 indicate that PTSD research has been focused on war trauma. The terms of therapy, e.g., exposure therapy, imaginal exposure, and prolonged exposure, listed in Table 3 suggest that therapies for PTSD are most related to exposure, as exposure therapy (ET) is one of the most recognized trauma-related therapies for symptom remission. ET involves graded exposure to situations leading to fear response, enabling an individual to become desensitized to fear cues [44]. ET integrated with VR, namely virtual reality exposure therapy (VRET), is considered an alternative therapy to allow patients to experience a presence in a computer-generated, three-dimensional environment that is immersive and interactive, to help with minimizing avoidance behavior strategies and promote the emotional participation of the patients [45].

There have been various case studies [46,47,48,49,50,51,52,53] that provided preliminary evidence for the efficacy of VR-aided therapy to treat PTSD. In addition, an early control study was conducted in 2010 [54] and followed by several randomized controlled trials focusing on the differences that VR makes [55,56,57,58]; these studies stressed the importance of experimental methods that influence the results. Further, there were a number of review studies, such as literature reviews [45,59,60,61,62,63,64], systematic reviews [65], and meta-analyses [66,67]. The most common topic was found to be the outcome of VR-aided therapy while exploring certain issues, e.g., Botella et al. [61] paying attention to the adequacy and acceptability of VR treatment, Deng et al. [67] aiming to identify potential variables, and Motraghi et al. [63] reviewing methodologies.

As the outcome terms significant reduction and effective treatment suggest, numerous studies have reported that VR is useful for PTSD. For example, Wood et al. found that VRET can alleviate the measurable physiological responses and PTSD symptoms of veterans who participated in the war on terror. A similar effect was found in the therapy of active service members (both males and females) [48,50,68]. Moreover, Difede et al. [51] claimed that VR is effective for survivors of terrorist attacks, and it can help those who cannot engage with traditional imagination therapy. Furthermore, Walsh et al. [69] developed an exposure program comprising a virtual driving game and virtual environment, and they found that it was feasible to use such a computer-generated environment to treat PTSD for traffic accidents, even when the patient’s condition was complicated with depressive symptoms. Hence, the therapeutic effects of VR for various kinds of PTSD have been proven. Interestingly, engagement with VR has been claimed to improve the satisfaction of patients [52]. However, the meta-analysis of Kothgassner et al. [66] showed only moderately positive outcomes of VRET to treat PTSD, which could be explained by the difference of ability to immerse individuals.

The outcome term potential benefits in Table 3 show that VR could benefit therapies for PTSD. Rothbaurn et al. [46] reported that VR could scale up therapists’ choices, enable the creation of customized virtual environments, and establish shared experiences. MR makes it easier to transmit and control corresponding stimuli to patients and make them more actively participate in therapy, which will activate more traumatic memories to increase the possibility of eliminating conditional fear [65]. Therefore, VR-aided therapy may be helpful for PTSD patients who are resistant to traditional exposure. In addition, VR can maintain long-term contact with stimuli in ways that are safer and more feasible than other exposure therapies [70]. Further, VRET has not shown iatrogenic or negative results in which the curative effect remains unchanged in the long-term follow-up, and their recurrence rate is shallow (4.5%) [55]. Deng et al. [67] argued that VRET has a sustained therapeutic effect and does not have the so-called dependence or withdrawal reaction in drugs, as well as that there could be a positive correlation between VRET dose and efficacy response, which suggests that VR-aided therapy may benefit from frequent use.

#### 3.3.2. Anxiety and Fear Related Disorder (A&F)

Clinically, fear can be regarded as the response to a specific cue, while anxiety is a more long-lasting phenomenon that is not specific to overt cues; both are debilitating situations that affect a significant number of individuals [71]. Terms associated with A&F from Figure 6 are categorized in Table 4.

For A&F therapies, VR is as effective in inducing emotional responses as reality, and its application is extremely valuable in exposure treatment [72], as reflected by the therapy terms vivo exposure and VR exposure in Table 4. However, there have been several studies on VR-based cognitive behavior therapy (VR-CBT) [73,74,75,76,77] since it is the standard evidence-based psychological treatment for anxiety disorders [78]. Some other therapies also appeared in this field, including mindfulness therapy [79] and talking cure [80]. Additionally, some studies highlighted game elements in the therapy process [80,81,82,83].

VR for A&F is well-studied. Some case studies [80,84,85,86] have provided qualitative evidence for the efficiency of VR-aided therapy. Additional controlled trials [87,88,89,90,91,92,93,94,95] compared the effects of virtual exposure and in vivo exposure, and two special studies [88,91] compared the differences between self-help virtual therapy and therapist-guided therapy. There have been a number of secondary studies, including literature reviews [72,78,96,97,98,99,100,101,102,103,104], systematic reviews [105,106], and meta-analyses [107,108,109,110,111,112,113,114,115,116,117,118], that evaluated the efficacy of VR. Moreover, various studies [98,106,111,115,118] have explored the mechanism of VR-aided therapy by reviewing moderated factors of outcome, such as presence, therapy alliance, cognition process, and therapy duration. Anderson and Molloy [96] shed light on the safety, technical advantages, and acceptance of VR, and further studies [99,104,105,114] identified the main challenges of promoting VR and assessed the regularity of common applications. Furthermore, in terms of measurement approach, some studies used qualitative self-report measurements [73,87,115] and others employed ecological measurements [119], such as heart rate [120,121] and brain metabolism [122].

The outcome terms effective treatment and significant reduction in Table 4 echo the effectiveness of VR to some extent. Several studies reported that VR-based therapy could provide a long-term therapeutic effect for anxiety disorders. In Wallach et al.’s [74] randomized clinical trial, VR-CBT was equally helpful as traditional CBT for public speaking anxiety and significantly more effective than waiting list control in anxiety reduction and subject’s self-rating of anxiety during a behavioral task. A follow-up study showed that the improvement of symptoms was maintained for one year [75]. Similarly, VRET is believed to have a comparable effectiveness to exposure group therapy for treating social fears, and refinement has been maintained for one year [93]. In addition, VR can exert functions in the treatment of phobias. The virtual bungee jumping environment developed by Jang et al. [123] provided a preliminary illustration of the effectiveness of VRET in treating acrophobia. Triscari et al. [73] highlighted that VR-based treatment can significantly improve the symptoms of flight phobia, and the effect could last for one year when associated with high measurement scores. The behavior and attitudes of the patients to actual flight were changed after the therapy. Interestingly, VR is also helpful for phobias of small animals, such as the game developed by Lindner et al. [81,82] for spider phobia.

However, the outcomes of VR-based therapy for various medical conditions are different. In some specific disorders, VR can achieve outstanding results. The meta-analysis of a study by Cardo et al. [113] showed that VRET was more effective than any other traditional evidence-based therapy for fear of flying. However, for more general conditions, Carl et al. [108] concluded that VRET is just as helpful as in vivo exposure for many anxiety-related diseases, although it exerted a more significant influence range than the control group and the influence was only medium or large when treating specific phobias, sadness and performance anxiety, post-traumatic stress disorder, and Parkinson’s disease. In contrast, VR is inferior to traditional therapy in some conditions. For example, Wechsler et al. [109] demonstrated that the therapeutic outcome of VRET for agoraphobia is significantly lower than the use of in vivo exposure. Additionally, Jiang et al. [124] argued that VR cannot be a standard treatment for blood-injection-injury phobias since it has no impact on the confidence of patient to deal with the fear situation. Despite the discrepancy of effects, VRET is regarded as an acceptable and effective substitute for in vivo exposure, and it is believed that it will improve with advancements of technology and procedure [109].

Due to differences in effectiveness, research on VR for therapy is suggested to determine practical enhancements for anxiety, phobias, and clinical predictors of improvements [118]. Most studies have focused on the sense of presence, which refers to the awareness of being in an environment, either real or virtual [125]. Presence has been found to influence the anxiety experience of a virtual environment, and it has a relationship with fear factors within VR. However, there is a lack of evidence to support an association between presence and therapeutic effects, which means that presence seems to be a necessary condition of the therapeutic effect but not strong enough to influence the therapy results [115,126]. A study based on self-reported results regarding public speaking anxiety [127] suggested that maximizing presence may only increase the experienced fear, while maximizing involvement, or attentional focus, may lead to better treatment responses. It seems that presence is a requirement for a successful outcome because it induces anxiety and fear [105]. In a study of VR-CBT [92], the contributions of immersion and presence to therapy results were specious, though the study results supported a significant correlation between patient expectations and most changes in therapeutic effects, i.e., the work alliance evaluated by patients had a medium effect in explaining patients’ rational and irrational modifications and changes in anxiety symptoms and therapist’s performance also significantly impacted the changes of anxiety symptoms. Hence, how presence affects the experiences of patients and therapy results is debatable, while other factors, such as participation, the expectation of patients, and work alliance, can influence the outcome of therapy to a different extent.

The outcome terms, e.g., low cost, availability, and wide range, in Table 4 convey the benefits of VR in A&F. Cost-effectiveness is a remarkable feature of therapeutic VR, especially in the treatment for fear of flying, since VR can generate more gradual therapy settings (sequence and intensity) and efficiently create exceptional exposure (different flight destinations, crew members, and weather conditions) that can be endlessly reused in therapy [117]. Malbos et al. [83] claimed that VRET is a more economical treatment for claustrophobia in comparison to in vivo exposure. As the cost of a complete VR system has been substantially reduced, the technology has become more available [75]. Since it allows for individualized, progressive, controllable, and immersive exposure and is accessible for therapists and patients, the attitudes of therapists and patients toward VR have changed are no longer the main obstacle to implement next-generation VR in routine clinical practice [99,128]. Although, the compliance of VR-based therapy is superior to traditional treatment, the VR-based therapy does not show noticeable adverse effects compared with in vivo exposure, and VR exposure and in vivo exposure therapy have shown similar loss rates [111]. Additionally, the deterioration rate of VR-based therapy is consistent with or lower than other treatments [110]. However, more knowledge is needed to promote VR for therapy, including regarding how to achieve greater clinic acceptability, better strategies for exposure, and the avoidance of reoccurrence [105].

#### 3.3.3. Diseases of the Nervous System (DNS)

Diseases of the nervous system include dyskinesia, neurocognitive impairment, cerebrovascular disease, and cerebral palsy [39]. Terms associated with DNS from Figure 6 are categorized in Table 5.

The motor function and balance of patients are the focuses of DNS research using medical condition terms, such as balance, motor, and gait. The therapy terms conventional therapy and virtual reality therapy refer to the two most discussed therapies in the DNS field. VR-based therapies are mainly integrated with conventional therapy, such as movement [129,130,131], serious game [132,133,134,135,136,137], mirror [138,139,140], and gesture therapy [141,142].

As shown in Table 5, the main research method used for VR for DNS is the randomized controlled trial [129,143,144,145,146,147], with which most studies compared VR-aided therapy with conventional rehabilitation and one study [147] assessed the impact of adding psychological training. In addition, systematic review [133,148,149] and meta-analysis [135,150,151] studies of the field have concerned the evaluation of VR and conventional rehabilitation therapies, while literature reviews have provided some information about the benefits of VR [152,153]. In terms of the measurement method, the Berg, Fugl-Meyer, and stoke influence scales are the most used in balance, function, and quality of life assessments [136].

An advanced method for DNS research is functional magnetic resonance imaging (fMRI), which can be used to explore the neuroplasticity of neural rehabilitation patients. Orakpo et al. [18] argued that the promising behavioral improvements demonstrated in clinical trials must be associated with an understanding of cortical and subcortical changes that form the biological basis of rehabilitation. The compatibility between VR and such imaging technology has enabled researchers to present multimodal stimuli with high ecological effectiveness while recording changes in brain activity, which is also beneficial for therapists [154]. The observed neural reorganization results have provided convincing evidence for the effectiveness of VR rehabilitation. For example, You et al. [155] used fMRI to detect the neuroplasticity changes in the smooth muscle cells of an eight-year-old cerebral palsy patient during VR therapy via three different motor games, and these changes seemed to be closely related to the enhancement of age-appropriate motor skills in the affected limbs, which supported the effectiveness of VR to treat children with hemiplegic cerebral palsy based on the principle of neuroplasticity. Orihuela-Espina et al. [142] conducted a study on upper limb hemiplegia caused by stroke in order to quantify the neural plasticity changes given to patients by VR gesture therapy, of which the prefrontal cortex and cerebellum activities have been found to be the driving forces for rehabilitation. However, the impacts on neuroplasticity of VR-based therapy and conventional therapy have been found to be similar but different. This may be because visual and tactile feedback enhancement affect the high-order somatosensory and visual–motor areas when using VR [156].

The terms in Table 5 indicate that VR is effective for DNS. For example, Okmen et al. [145] studied children with cerebral palsy and found that VR significantly contributed to the success of treatment to improve their motor function. In addition, VR rehabilitation has been claimed to dramatically improve the balance and gait of Parkinson’s patients compared to traditional physical therapy [143]. Further, Mohammadi et al. [151] systematically reviewed comparative studies on the efficacy of VR-enhanced conventional therapy and conventional therapy, and they showed that VR-enhanced traditional therapy was more effective in improving the balance of post-stroke patients. The outcome terms positive effect and significant improvement in Table 5 also indicate that VR is an effective tool for DNS. However, further high-quality research is needed, and there are still challenges such as the content of intervention measures and the generality of research results at different stages of the disease, thus suggesting a lack of clarity regarding the most effective type associated with the time, setting, and duration of VR-based therapy [157,158].

The outcome terms motivation, participation, and mobility may indicate that VR is beneficial in DNS rehabilitation. VR can improve the motivation and emotions of patients. For instance, in addition to improving the postoperative balance ability, VR could significantly enhance patients’ motivation, participation, and cooperation level in Sharar et al.’s study [159] on children with cerebral palsy. Additionally, Wille et al. [134] highlighted that VR, as a part of a pediatric interactive therapy system for dyskinesia, can facilitate the improvement of patient participation be giving them a greater freedom of self-control, reducing the cost of therapy by using a group therapy environment, and helping with the objective evaluation of the progress of a patient’s condition through game score and difficulty level settings. This also shows that VR is customizable. VR can provide a functional, purposeful, and motivating context for rehabilitation and therapies, is easily graded, and is easily documented [160]. Therefore, VR allows the patients to enjoy customized therapies. For therapists, VR can aid in the observation of the conditions of patients, which is useful for research work. Moreover, the application of VR for DNS treatment has great mobility. Such an intervention could be conducted in-home, which can become a family-based complementary treatment [139], and it will become a fundamental aspect of personalized therapy and telemedicine [153].

#### 3.3.4. Pain Management (PM)

Uncontrolled pain has a universal and potentially negative effect on quality of life [161]. Brennan et al. [162] argued that PM is supported to be a fundamental human right. Formal pain management comprises the improvement of understanding of pain generation mechanisms and the use of analgesic drugs [163], and there are more alternative therapies than ever. The medical condition term burn patient may denote that burning pain is the focus of VR in PM. Terms associated with PM from Figure 6 are categorized in Table 6.

The therapy terms immersive virtual reality and distraction are shown in Table 6 and suggest immersive VR can be used as a distraction therapy. For instance, a study by Huang et al. [164] provided VR equipment to subjects suffering from pain and immersed them in a virtual landscape trip. In addition, VR can be used as assistance to psychopathology [165] or physical therapy [166].

The authors of most studies used case studies [167,168,169,170] to explore the use of VR, and several studies used randomized controlled trials [164,166,171,172] to find differences in VR effects. Interestingly, there was also a meta-analysis [173] of the analgesic effects of VR in burn patients undergoing dressing changes in physical therapy.

There is some evidence supporting the application of VR in PM. For example, Hoffman et al. [40] conducted a survey on physical therapy integrated with VR for adult burn patients, and VR was found to help reduce pain to a statistically significant extent according to the self-reported measurements. Pain reduction also appeared in the study of Schmitt et al. [166], where VR was used to conduct a distraction therapy for pediatric burns and was claimed to remarkably improve the interest level of patients. Additionally, VR has been used in a wide range of medical conditions of pain and achieved positive results. For example, Hoffman et al.’s [170] preliminary evidence showed that VR can be used as an auxiliary and non-drug pain relief technique for multiple blunt traumas. Moreover, a review [173] showed moderate evidence for VR treatment to reduce both pain and functional damage in patients with acute pain. Furthermore, the effect of VR to alleviate pain is relatively stable among various populations, since Sharar et al. [174] showed that pain relief is not influenced by age, gender, and race, as well as that the effectiveness of VR is irrelevant to the sense of presence. Meanwhile, VR’s analgesic effect does not become ineffective with repeated use [169]. Nevertheless, VR cannot replace traditional analgesic methods. For instance, there is no evidence for the continued effectiveness of VR for chronic pain, although it has short-term effects, suggesting that the use of non-opioid drugs to treat chronic pain is infeasible [167]. Likewise, in a study exploring the impact of intravenous infusion on the self-sedation needs of patients undergoing artificial joint replacement under regional anesthesia, although immersive VR was well-tolerated, it did not reduce the overall sedation needs of patients undergoing joint replacement surgery [164].

VR has various advantages in PM, as well as great acceptance. Ford et al. [175] evaluate the views of key stakeholders (i.e., patients and providers) on the feasibility, acceptability, and effectiveness of using low-cost VR technology in routine burn care, and the quantitative and qualitative results unanimously supported applying low-cost VR technology in burn clinics. Their study also highlighted that VR is cost-effective for pain dispersion. Currently, highly immersive VR is available and affordable, and more patients can use it for pain control, potentially at home [168]. VR is practical for various other uses, too. More patient groups, such as those with chronic back pain [176], fibromyalgia [177], and cancer [165], can receive VR treatment.

## 4. Discussion

In this paper, VR-aided therapy has been investigated, and a BCM was created to visually map out the research landscape in the field. Four major areas were identified: A&F, PTSD, DNS, and PM. A&F and PTSD are related to psychology and psychiatry, while DNS and PM are associated with neurology and rehabilitation. The network visualization of VR-aided therapy revealed that recent studies have mainly been focused on A&F and PTSD, which is in line with Thurner et al.’s [178] study that indicated that psychology and psychiatry are the medical fields where VR studies are growing, thus suggesting that VR has exceptional strength in these fields. Although the importance of rehabilitation is not highlighted in this paper because there were few related studies shown in the network visualization, a previous study [179] claimed that VR, when used as a tool for evaluation and treatment, has drawn attention in this area.

The application of VR in HC from the perspective of therapy also has been investigated, showing that VR for use in therapy is a relatively emerging field with no standard approach. David et al. [78] claimed that VR is not a new therapy sect but a tool to facilitate traditional therapy, although it has numerous unique characteristics. Accordingly, there are considerable differences between VR-based therapies [170], which may include interventions from ET, CBT, movement therapy, game, mirror therapy, gesture therapy, and distraction therapy. It can be inferred from the current situation that VR has excellent scalability and the potential to integrate with more novel therapies. For example, Hacmun et al. [180] suggested that art therapy with VR could improve medical and HC services.

Moreover, methods for research on therapeutic VR have been investigated in this paper, revealing that case studies and randomized controlled trials are the most frequently mentioned methods. However, there are difficulties in further systematic reviews or meta-analyses, as Botella et al. [61] argued that some studies have failed to use VRET following the clinical guidelines of PTSD evidence-based intervention. In addition, as mentioned in Section 3.3.1, the influence of experimental methods and measurements is a considerable reason for the observed disparity of results. Accordingly, as Birckhead et al. [181] stressed, it is necessary to first build consensus regarding the scientific framework for VR therapy development and evaluation and then provide a methodological framework of clinical research according to the opinions of an international working group. 

The overall results of the therapeutic effect of VR are optimistic and can be used for evidence-based HC design. An interesting finding is that the psychological effects of VR seem to be beneficial to both mental and physical health. For example, the integration of VR with traditional rehabilitation techniques can improve psychological adaptation, thus making it an essential contribution to cerebral palsy therapy [182]. In addition, Park and Park [147] highlighted that the incorporated psychotherapy of VR could provide more remarkable improvements during the therapy process for upper extremity rehabilitation. It seems that the psychological benefits may be a mediating factor of therapeutic effects, improving patients’ quality of life. As pointed out in a mental health report issued by Department of Health, the United Kingdom [183], good mental health is paramount to physical health and other outcomes. Unfortunately, the results of network visualization in this paper show that the link between physical and psychological research is weak, which may require the further introduction of more medical theories such as psychosomatic medicine. The psychosomatic medicine is a branch concerning the interaction between psychosocial and biological factors in the process of disease [184]. In the future research of therapeutic VR, studies should not ignore the impact of psychological effects on final therapy outcomes, and HC service providers should better learn how psychological impacts improve HC services.

Furthermore, this paper indicates that VR is advanced in customization, compliance, cost, accessibility, motivation, and convenience. A key question is how to use these features to achieve a better outcome. Several successful therapies have applied game elements to the process, resulting in serious games [69,81,82,136,155]. These methods can be called gamification, a conceptual framework to apply game elements and techniques to optimize the process in a non-game context, and they can motivate players to perform challenging tasks with game mechanics, dynamics, and components [185]. In the context of HC, Pereira et al. [185] noted that gamification is beneficial for the users’ emotional experiences, sense of identity and social positioning, cognition, social skills, and psychomotor skills. It seems that VR with gamification may maximize the value of therapy; VR provides appropriate personalized simulation, adjustable stimulation, and repeatable and multi-person participation, and gamification can improve motivation, guide learning, change cognition, and provide accurate data [186,187,188,189]. Udara and Alwis [188] hypothesized that the extensive use of augmented reality and VR in such gamification solutions will be more visible in the future; they could comprise a feasible way to realize the vision of Health 4.0. Hence, researchers could attempt to add more game elements into VR therapy and clarify their practical effects, as done by Muangsrinoon and Boonbrahm [189], who studied the use of points, feedback, levels, leader boards, challenges, badges, avatars, competition, and co-operation in the use of VR for HC.

The identified four limited year ranges in two decades (2000 to year 2020), as shown in Figure 5, illustrating the progress of VR-aided therapy development from for treating PTSD to A&F, could be regarded as the early development (pre-life) stages of the Health Metaverse. Healthcare providers and patients can well communicate in the virtual world through gamification, which has shown a potential to monetize digital health integrated with other latest emerging technologies, such as Blockchain and Non-Fungible Token, for helping future consumers to actively manage their health and making smarter healthy decisions [190]. Indeed, the result of this paper is a map of pre-life of the Health Metaverse, as Chen and Zhang [191] highlighted the innovative use of medical VR that will be an important part of Health Metaverse. It is predictable that the arrival of Health Metaverse will be accelerated because of the technology integration and the epidemic. Therefore, future research could be devoted to explore the function of VR-aided therapy in Health Metaverse facilitating with achieving more sustainable and social significance to meet the feature of the metaverse [192].

Although VR can bring outstanding outcomes [6], including therapeutic effects and benefits, there are challenges for in the application of VR. Sharma et al. [193] stated that VR has technical, physical, privacy, behavioral, and investment risks, but the most significant problem is obtaining funds, which depends on the level of public acceptance. Additionally, the profit produced by the HC industry seems to be relatively low, which is not attractive to most game developers [189]. Moreover, the barriers and facilitators of VR in healthcare include technology developments, end-user capabilities, and clinical settings [194]. Furthermore, the gap of population diversity, such as the acceptance of elderly patients [195], needs more research [26]. Therefore, studies on therapeutic VR must consider more stakeholders, e.g., patients, therapists, developers, and designers, and more dimensions, e.g., production, service, and therapy circumstances. A possible solution could be the use of design research methods that can notably contribute to future HC. This could bring opportunities for health communication, prototyping, co-design, digital design, salutogenic design, and holistic design [196]. Some studies have mentioned design-based research, such as user-centered design [197], usability [198], and innovative methods [24]. The results of these studies encourage the development of design research in VR for therapy, which is a significant part of the HC system’s design.

## 5. Conclusions

The purpose of this study was to explore an overview and details of VR and therapy and to guide VR application in HC, which was achieved with the bibliometric analyses of publications from WoSc. The bibliometric analyses of articles and terms showed the potential to explore VR-aided therapy from the perspective of HC, which provides reliable macro knowledge since HC is a broad field and hard to summarize with other methods, such as literature reviews. In addition, a variety of visual maps can help healthcare stakeholders overcome the limitations of a single medical condition with multi-perspective insights, which could facilitate the development of VR-aided therapy and HC.

This paper makes the following contributions to the field. First, this study was the first attempt to use VOSviewer to conduct bibliometric analyses of VR and therapy from the perspective of WoSc, a widely used database for science and technology as well as medical and health domains, which objectively and visually shows research structures and research topics in contrast to traditional literature reviews. Secondly, this study was the first systematic investigation of the research status of VR-aided therapy incorporating articles of two decades (2000 to year 2020), which not only provides a panorama of this field but also shows the details of four major research areas, i.e., PTSD, A&F, DNS, and PM, including medical conditions, therapies, methods, and outcomes. This could bridge the gap of knowledge of VR and therapy, as well as inspire future studies. Thirdly, this paper presents a discussion of the use of VR to aid therapy from a holistic point of view, which provides the foundation for future studies on Health Metaverse.

This paper highlights that VR-aided therapy is effective for various medical conditions, and VR has advantages in customization, compliance, cost, accessibility, motivation, and convenience that highlight its potential in HC. These advantages enable VR technology to be integrated into a variety of therapies and help traditional therapies overcome the limitations of physical factors, which is important in the current context of the coronavirus disease 2019 (COVID-19) pandemic. Additionally, a VR environment can easily stimulate people’s emotional response because it is an excellent medium to combine psychotherapy and physical therapy and to provide more abundant patient data to HC professionals, which could improve the relationship between therapists and patients. Furthermore, VR can be integrated with emerging methods, such as gamification and user-centered design, to provide customized therapy services for patients and improve the quality and satisfaction of the healthcare system. Overall, these potentials of VR help achieve the vision of Health 4.0 and even future exciting Health Metaverse.

This paper offers instructive insights for HC stakeholders, particularly researchers and service providers, regarding the integration of more innovative therapies, psychological benefits, game elements, and design research. Further, this paper provokes questions that need further investigation regarding the standardization of research methods and the difficulties for VR application in the context of HC, among others. However, several limitations of this study need to be considered. In this paper, WoSc has been adopted for collecting data. Additionally, the authors used two types of publications, i.e., peer reviewed articles and reviews, to ensure the high quality of publications. In the future, more databases, e.g., PubMed, and types of publications, e.g., conferences papers, can be used in this area of study, which may provide updated knowledge regarding VR and therapy.

## Figures and Tables

**Table 1:** The representative articles of each cluster in Figure 2 for virtual reality (VR) aided therapy (devised by the authors).

Cluster	Author	Year	Research Method	Research Subject	Medical Condition	Therapy
1	Marco et al.	2013	Controlled study	Women	Eating disorders	Body image treatment using Virtual reality
	Riva et al.	2002	Controlled study	Adult women	Eating disorders	Multidimensional virtual reality treatment
	Ferrer-garcia et al.	2017	Randomized Controlled Comparison	Adults	Eating disorders	Virtual reality cue exposure therapy
2	Hoffman et al.	2000	Case study (self-report)	Adults	Burn pain	Virtual-reality-aided physical therapy
	Schmitt et al.	2011	Randomized, controlled trial	Teenagers	Burn pain	Virtual-reality-aided physical therapy
	Hoffman et al.	2014	Case study	Teenagers	Burn pain	Occupational therapy with virtual reality
3	Rizzo et al.	2005	SWOT analysis	Unclear	Rehabilitation	Unspecified
	Bohil et al.	2011	Literature review	Unclear	Neuroscience related	Unspecified
	Lohse et al.	2014	Systematic review	Unclear	Post-stroke	Custom built virtual environments and commercially available gaming systems.
4	Rothbaum et al.	2001	Open clinical trial	Vietnam combat veterans	Post-traumatic stress disorder	Virtual reality exposure therapy
	Rothbaum et al.	2014	Controlled randomized clinical trial	Iraq and Afghanistan veterans	Post-traumatic stress disorder	Virtual reality exposure with drugs
	Difede et al.	2007	Controlled trial	Civilians and disaster workers	Post-traumatic stress disorder	Virtual reality exposure therapy
5	Parsons et al.	2008	A meta-analysis	Unclear	Anxiety and specific phobias	Virtual reality exposure therapy
	Powers et al.	2008	A meta-analysis	Unclear	Anxiety and specific phobias	Virtual reality exposure therapy
	Rothbaum et al.	2006	Controlled clinical trial	Unclear	Fear of flying	Virtual reality exposure therapy
6	Opris et al.	2012	A meta-analysis	Unclear	Anxiety disorders	Virtual reality exposure therapy
	Morina et al.	2015	Case study	College students	Anxiety disorders	Virtual reality exposure therapy
	Carl et al.	2019	A meta-analysis	Unclear	Anxiety disorders	Virtual reality exposure therapy
7	Botella et al.	2017	Systematic review	Unclear	Phobias	Virtual reality exposure therapy
	Freeman et al.	2018	Randomized trial	Adults	Fear of heights	Virtual reality cognitive intervention
	Lindner et al.	2017	Literature review	Unclear	Anxiety disorders	Virtual reality exposure therapy

**Table 2:** The most frequent terms on the theme of VR and therapy in the Web of Science core collection (WoSc) database calculated by VOSviewer (devised by the authors).

Rank	Term	Occurrences
1	Virtual reality exposure therapy	94
2	Exposure therapy	46
3	Virtual reality therapy	43
4	Test	41
5	PTSD	38
6	Rehabilitation	35
7	Control group	33
8	Anxiety disorder	31
9	Post-traumatic stress disorder	28
10	Phobia	28

**Table 3:** Categorized terms associated with post-traumatic stress disorder from Figure 6 (devised by the authors).

Medical Condition	Therapy	Method	Outcome
IRAP, combat, veteran, active duty soldier, anxiety symptom, cognitive function, post-traumatic stress, PTSD, PTSD symptom, stress disorder	Course, exposure therapy, imaginal exposure, post-treatment, pretreatment, prolonged exposure, virtual reality exposure (VRE)	Case report	Engagement, frequency, immersion, potential benefit, significant change

**Table 4:** Categorized terms associated with anxiety and fear related disorder from Figure 6 (devised by the authors).

Medical Condition	Therapy	Method	Outcome
Avoidance, depressive symptom, distress, panic disorder, paranoia, phobia, public speaking anxiety, sad, social anxiety, social anxiety disorder, social phobia, social situation, specific phobia, spider	Post-treatment, pre-treatment, virtual reality cognitive, virtual reality exposure, vivo, vivo exposure, vivo exposure therapy, VR CBT, VR exposure, VR exposure therapy, VRET session	Control condition, heart rate, self-reporting	Availability, effect size, effective treatment, extent, low cost, relaxation, significant reduction, wide range

**Table 5:** Categorized terms associated with diseases of the nervous system (DNS) from Figure 6 (devised by the authors).

Medical Condition	Therapy	Method	Outcome
Adult, balance, cerebral palsy, child, children, gait, motor, motor function, motor rehabilitation, movement, stroke, upper limb	Conventional therapy, post intervention, VR therapy, VR group, VR intervention	Berg balance scale, control group, experimental group, Fugl Meyer assessment, randomized clinical trial, systematic review, meta-analysis, test	Duration, mobility, motivation, participation, performance, positive effect, significant improvement, significant result

**Table 6:** Categorized terms associated with pain management (PM) from Figure 6 (devised by the authors).

Medical Condition	Therapy	Method	Outcome
Burn patient, chronic, female, pain	Attention, distraction, exercise, immersive virtual reality, physical therapy, VR session	Case study	Average, preliminary evidence, range, side effect

## Data Availability

Publicly available datasets were analyzed in this study. These data can be found here: https://login.webofknowledge.com/ (accessed on 23 August 2021).
